# The impact of antenatal and postnatal indoor air pollution or tobacco smoke exposure on lung function at 3 years in an African birth cohort

**DOI:** 10.1111/resp.14576

**Published:** 2023-08-17

**Authors:** S. Chaya, A. Vanker, K. Brittain, R. MacGinty, C. Jacobs, Z. Hantos, H. J. Zar, D. M. Gray

**Affiliations:** ^1^ Department of Paediatrics and Child Health, Red Cross War Memorial Children's Hospital and SA‐MRC Unit on Child and Adolescent Health University of Cape Town Cape Town South Africa; ^2^ Department of Anaesthesiology and Intensive Therapy Semmelweis University Budapest Hungary

**Keywords:** childhood lung function, environmental tobacco smoke, indoor air pollution, multiple breath washout, oscillometry, tidal breathing flow volume loop

## Abstract

**Background and Objective:**

Indoor air pollution (IAP) and tobacco smoke exposure (ETS) are global health concerns contributing to the burden of childhood respiratory disease. Studies assessing the effects of IAP and ETS in preschool children are limited. We assessed the impact of antenatal and postnatal IAP and ETS exposure on lung function in a South African birth cohort, the Drakenstein Child Health Study.

**Methods:**

Antenatally enrolled mother–child pairs were followed from birth. Lung function measurements (oscillometry, multiple breath washout and tidal breathing) were performed at 6 weeks and 3 years. Quantitative antenatal and postnatal IAP (particulate matter [PM_10_], volatile organic compounds [VOC]) and ETS exposures were measured. Linear regression models explored the effects of antenatal and postnatal exposures on lung function at 3 years.

**Results:**

Five hundred eighty‐four children had successful lung function testing, mean (SD) age of 37.3 (0.7) months. Exposure to antenatal PM_10_ was associated with a decreased lung clearance index (*p* < 0.01) and postnatally an increase in the difference between resistance at end expiration (ReE) and inspiration (*p* = 0.05) and decrease in tidal volume (*p* = 0.06). Exposure to antenatal VOC was associated with an increase in functional residual capacity (*p* = 0.04) and a decrease in time of expiration over total breath time (*t*_E_/*t*_TOT_) (*p* = 0.03) and postnatally an increase in respiratory rate (*p* = 0.05). High ETS exposure postnatally was associated with an increase in ReE (*p* = 0.03).

**Conclusion:**

Antenatal and postnatal IAP and ETS exposures were associated with impairment in lung function at 3 years. Strengthened efforts to reduce IAP and ETS exposure are needed.

## Abstract 2

Indoor air pollution (IAP) contributes to the high burden of childhood respiratory illness. We performed comprehensive lung function testing, showing both antenatal and postnatal IAP and environmental tobacco smoke exposure to be associated with impairment in lung function at 3‐years of age. This highlights the need for public health awareness and initiatives.

## INTRODUCTION

Air pollution is a global health concern contributing to the high burden of respiratory disease. Low and middle‐income countries (LMICs) are disproportionately affected due to increased reliance on unclean fuel sources such as fossil fuels for household energy.^1^ Women and young children are particularly vulnerable to the harmful effects of indoor air pollution (IAP) as they spend more time indoors cooking, often with poor ventilation.^2^ IAP and environmental tobacco smoke (ETS) are well described risk factors for childhood respiratory disease and impaired lung function.^3^ Solid and alternate fuels emit a mixture of pollutants including particulate matter (PM) and volatile organic compounds (VOCs), all of which threaten respiratory health.^3^, ^4^

In utero exposure to each of ETS and IAP is associated with poor birth outcomes, reduced lung function in infancy through to adulthood and increased childhood respiratory disease.^3^, ^5^, ^6^, ^7^ We have previously described that in utero exposure to ETS and household benzene reduces lung function 4–6 weeks after birth in the Drakenstein Child Health Study (DCHS), a South African birth cohort study.^6^, ^8^, ^9^

In addition, postnatal exposures have been associated with decreased lung function in adults and older children.^10^ In childhood postnatal exposures have been associated with a decrease in spirometry lung volumes and an increase in resistance and decrease in reactance with oscillometry measurements.^10^, ^11^, ^12^, ^13^

However, there are limited data from longitudinal cohorts that measure both ante‐ and postnatal exposure. Many studies to date are retrospective and not conducted with preschool children. A better understanding of the impact of IAP and ETS on early lung development, particularly in communities with several exposures, is needed to develop targeted preventive strategies.

This study aimed to assess the impact of antenatal and early‐life IAP and ETS exposures on lung function at 3‐years of age in the DCHS cohort.

## METHODS

The DCHS is a prospective birth cohort study of mother–child pairs enrolled from March 2012 to March 2015. This study site is in Paarl, a peri‐urban, low socioeconomic community approximately 60 km outside Cape Town, South Africa. Participants were enrolled at two primary health care clinics.^14^ Consecutive consenting mothers were enrolled during the second trimester of pregnancy, and mother–child pairs were followed. Study visits synchronized with the national immunization program, and included visits at 6, 10 and 14 weeks, at 6 and 9 months, and then every 6 months from 12 months onward detailed in the Supporting Information.^14^, ^15^ This analysis uses prospectively collected data from 6 weeks through to 3 years of age between July 2012 and October 2015. All children with successful lung function measurements at 3 years were included, although we excluded children born before 32 weeks of gestation and those living with HIV as they generally have poorer lung function for reasons not related to exposure to IAP or tobacco smoke. The study was approved by the University of Cape Town Faculty of Health Sciences human research ethics committee (048/2020; 082/2018; 423/2012).

### Clinical data collection

Socioeconomic status (SES) was assessed antenatally using a validated score derived from employment status, maternal educational attainment, household income, assets and market access.^16^ Gestational age at birth was calculated based primarily on antenatal ultrasound performed in the second trimester.^17^

Anthropometry was measured at all study visits, and weight and length were converted to *z*‐scores using Anthro software (WHO, Geneva, Switzerland).^18^ Weight‐for‐age *z*‐scores (WFA), height‐for‐age *z*‐scores (HFA) and body mass index (BMI) for age *z*‐scores were calculated. WFA < −2 and HFA < −2 were classified as underweight and stunted respectively.

Active surveillance for lower respiratory tract infection (LRTI) was done from birth through 3 years of age; LRTI was defined according to World Health Organization (WHO) criteria with findings confirmed by a trained study doctor or nurse.^19^

### IAP and ETS exposures

Home visits were conducted antenatally (28–32 weeks gestation) and postnatally (4–6 months of age) and PM_10_ and VOCs, benzene and toluene, measured. For PM_10_, a personal air sampling pump (AirChek 52®; SKC, Eighty Four, PA, USA) was left in the home for 24 h and a 24‐h average was obtained. Passive diffusion tubes (Markes® thermal desorption tubes; Llantrisant, UK) which measured VOC were placed in the homes for 2 weeks and a 2‐week average was obtained, as previously described.^20^ Antenatal ETS exposure was measured using maternal urine cotinine collected at an antenatal visit (28–32 weeks gestation) and at birth using the Immulite^R^ 1000 Nicotine Metabolite Kit (Siemens Medical Solutions DiagnosticsR, Glyn Rhonwy, UK) quantitative urine cotinine test.^21^ The highest measured value was used to assign antenatal exposure. Postnatal ETS exposure was determined using the highest infant urine cotinine measurement, done at 6 weeks, and yearly until 3 years of age.^20^

Exposure levels for each pollutant were defined using the South African National Ambient Air Quality Standards.^22^ Levels for benzene, toluene or PM_10_ were categorized as above threshold if the level was more than 5, 240 or 40 μg/m^3^, respectively.^20^ ETS exposure was quantified as no exposure (urine cotinine level <10 ng/mL), moderate (10–499 ng/mL) or high (≥500 ng/mL). Details of IAP measurement and ETS exposure methodology have been previously published and are summarized in the Supporting Information Table S1.^20^

### Lung function testing

All participants had lung function measurements at 6 weeks and 3 years of age but not within 4 weeks of a respiratory illness. Lung function measurements included intra‐breath oscillometry measuring respiratory impedance (Zrs) (resistance and reactance); tidal breathing flow volume loops (TBFVLs) with measures including tidal volume (TV), respiratory rate (RR) and expiratory flow ratios (ratio of time of expiration to total time [*t*_E_/*t*_TOT_] and ratio of time of peak total expiratory flow to time of expiration [*t*_PTEF_/*t*_E_]); and multiple breath washout (MBW) measuring the functional residual capacity (FRC) and the lung clearance index (LCI). All tests were done by the same team which included a respiratory technologist, a nurse and a paediatric pulmonologist. All testing followed international consensus guidelines.^9^, ^23^

Measurements at 6 weeks were performed in unsedated infants during quiet sleep as previously described.^9^ Intra‐breath oscillometry was performed with custom‐built wavetube equipment (University of Szeged, Hungary) using a 16‐Hz signal.^24^ The measurements of impedance were made in the supine posture, with the head supported in a neutral position, via a facemask and filter. Technically acceptable 30s recordings were collected. Recordings were excluded if they contained breath holds, cries, irregular breathing or leaks around the face mask.^8^ The intra‐breath oscillometry measures included were the resistance at end‐expiration (ReE) and at end inspiration (ReI), reactance at end‐expiration (XeE) and end inspiration (XeI), and the tidal changes ReE‐ReI (ΔR) and XeE‐XeI (ΔX). Mean resistance (Rmean) and reactance (*X*mean) for the whole breathing periods were also calculated. TBFVL and MBW measurements were collected using the Exhalyzer D with ultrasonic flow meter (Ecomedics, Duernten, Switzerland) and analysed with specialized analysis software (WBreath V.3.28.0; NDD Medizintechnik, Zurich, Switzerland). MBW was done using 4% sulfur‐hexafluoride (SF6) as a tracer gas.

At 3 years, lung function tests were performed in awake children. Oscillometry testing was completed using a custom‐made oscillometry system (INCIRCLE wavetube system, University of Szeged, Hungary).^25^ Measurement of one 16‐s epoch with 10 Hz oscillation frequency was recorded and repeated if necessary to obtain a minimum of five regular breaths, without any artefacts (vocal cord noise, apnoea, irregular breathing pattern, glottic closure, leak or sighs). Tests were conducted with the child sitting comfortably, nose occluded, with the cheeks firmly supported and breathing through a mouthpiece and filter. TBFVL and MBW measurements were collected using Ecomedics Exhalyzer D, Duernten, Switzerland and analysed with specialized analysis software (Spiroware 3.2.1, Zurich, Switzerland). MBW was measured during tidal breathing using inert nitrogen with 100% oxygen washout. Tests were performed with the child sitting and breathing comfortably through a size 2 silicone facemask (Laerdal) and filter (Gibeck Humid‐Vent Filter; Perak, Malaysia). The dead space of the mask was determined by water displacement.

### Statistical analysis

Data were analysed using Stata 14 (StataCorp Inc., College Station, TX). Child characteristics and exposures to IAP and ETS were summarized and compared between children included versus those excluded from analysis using Wilcoxon rank sum (Mann–Whitney) tests for continuous variables and chi‐square and Fisher's exact tests for categorical variables. A Pearson's correlation matrix was used to assess the association between each of antenatal and postnatal PM_10_, benzene, toluene and ETS. The independent effect of both antenatal and postnatal exposure to IAP and ETS on lung function measures at 3 years was examined using linear regression models adjusted for potential confounders. Confounders were selected a priori based on a directed acyclic graph (DAG) and included enrolment site, sex, SES, BMI *z*‐score at the time of testing, and ≥1 episode of LRTI prior to testing (Supporting Information Figure S1). Supplementary models explored the impact exposure to IAP and ETS at 3 years adjusted for these a priori selected confounders as well as lung function measured at 6 weeks, to assess the impact of these postnatal exposures adjusted for poor early‐life lung function.

For VOC, we combined benzene and toluene as these exposures were significantly correlated and are by‐products of the same alternate fuel combustion.

## RESULTS

**FIGURE 1:**
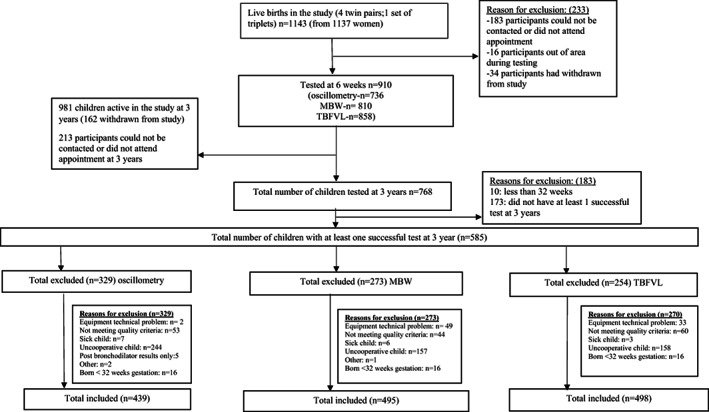
Description of all children in the cohort. MBW, multiple breath wash‐out; TBFVL, tidal breathing flow volume loop.

Of 768 children eligible for testing at 3 years, 584 (76%) with ≥1 successful lung function measurement (TBFVL, oscillometry and/or MBW) were included. Details of reasons for unsuccessful testing are shown in Figure 1. Compared to children excluded from analysis, those included were significantly less likely to be HIV‐exposed and to live in informal housing, and more likely to have antenatal ETS exposure (Supporting Information Table S2).

The mean age (SD) of the 584 children included was 37.3 (0.7) months (Table 1). Of these children, 49% were male, 13% were born late preterm (32–37 weeks), 19% were HIV‐exposed but uninfected, 7% were underweight, and 20% were stunted. Episodes of LRTI were common, with 49% experiencing ≥1 episode during the first 3 years of life (mean age at first episode: 8.6 [8.4 months]) of which 52% had recurrent LRTI.

**TABLE 1:** Characteristics of children with at least one successful test at 3 years of age.

	*n* (%)
Number of children	584
Mean (SD) age in months	37.3 (0.7)
Male sex	289 (49%)
Enrolment site	
Mbekweni	288 (49%)
TC Newman	296 (51%)
Born pre‐term (≥32 and <37 weeks gestation)	74 (13%)
HIV‐exposed	110 (19%)
Ever breastfed	477 (82%)
Median [IQR] months of breastfeeding	8.0 [2.0, 23.9]
Ever exclusively breastfed	342 (59%)
Median [IQR] months of exclusive breastfeeding	1.6 [0.7, 3.2]
Mean (SD) weight *z*‐score	−0.4 (1.1)
Underweight	42 (7%)
Mean (SD) height *z*‐score	−1.1 (1.1)
Stunted	117 (20%)
Mean (SD) BMI *z*‐score	0.4 (1.2)
Previous LRTI	284 (49%)
Recurrent LRTI	147 (25%)
Mean (SD) age of first LRTI in months (*n* = 284)	8.6 (8.4)
*Antenatal exposure to indoor air pollutants above ambient standards*	
PM_10_ (*n* = 406)	179 (44%)
Benzene (*n* = 393)	170 (43%)
Toluene (*n* = 393)	33 (8%)
Benzene and/or Toluene (*n* = 393)	171 (44%)
*Exposure to Antenatal tobacco smoke*	
Antenatal exposure (*n* = 566):	
Moderate exposure	262 (46%)
High exposure	202 (36%)
*Postnatal exposure to indoor air pollutants above ambient standards*	
PM_10_ (*n* = 285)	103 (36%)
Benzene (*n* = 257)	83 (32%)
Toluene (*n* = 257)	24 (9%)
Benzene and/or Toluene (*n* = 257)	83 (32%)
*Exposure to postnatal tobacco smoke*	
Postnatal exposure (*n* = 507):	
Moderate exposure	339 (67%)
High exposure	45 (9%)

*Note*: Underweight: weight‐for‐age *z*‐scores < −2. Stunted: height‐for‐age *z*‐scores < −2.

Abbreviations: LRTI, lower respiratory tract infection; PM_10_, particulate matter size 10 μg/m^3^.

Average lung function values at 6 weeks and 3 years of age are shown in the Supporting Information Table S3.

### Environmental exposures

Antenatal exposure to PM_10_ and VOC above ambient standards occurred in 44% of children, and postnatal exposure in over one‐third (Table 1). Antenatal ETS exposure occurred in 82% of children (moderate exposure in 46%; high exposure in 36%), and postnatal exposure in 76% (moderate exposure: 67%; high exposure: 9%). There was a strong correlation between exposures to antenatal benzene and antenatal toluene, as well as between postnatal benzene and postnatal toluene, but there was no correlation between antenatal and postnatal exposures. Strong correlations were however noted between antenatal and postnatal ETS exposure (Supporting Information Table S4).

### Impact of IAP and ETS on lung function at 3 years

#### PM_10_

The impact of PM_10_ on lung function at 3 years is presented in Table 2. In unadjusted analyses, antenatal PM_10_ exposure above threshold was associated with decreased LCI (*p* = 0.009), and postnatal exposure was associated with decreased RR (*p* = 0.062) and increased XeI (*p* = 0.067). In adjusted analyses, the association between antenatal exposure and decreased LCI persisted (*p* = 0.006), and antenatal exposure was associated with decreased tidal volume (*p* = 0.067). In addition, postnatal PM_10_ exposure was associated with increased ΔR (*p* = 0.052). After additional adjustment for 6‐week lung function, no significant associations with postnatal PM_10_ exposure were observed (Supporting Information Table S5).

**TABLE 2:** Impact of ante‐ and postnatal exposure to PM_10_ above ambient standards on lung function at 3 years of age.

	Unadjusted models			Adjusted models		
	*n*	*β* [95% CI]	*p*‐value	*n*	*β* [95% CI]	*p‐*value
ReE (hPa s L^−1^)						
Antenatal exposure	299	0.22 [−0.42, 0.87]	0.499	152	0.49 [−0.42, 1.40]	0.287
Postnatal exposure	203	0.31 [−0.51, 1.12]	0.457		0.60 [−0.33, 1.53]	0.205
XeE (hPa s L^−1^)						
Antenatal exposure	299	−0.13 [−0.53, 0.27]	0.518	152	−0.02 [−0.62, 0.57]	0.943
Postnatal exposure	203	0.20 [−0.31, 0.71]	0.435		0.00 [−0.61, 0.61]	0.998
ReI (hPa s L^−1^)						
Antenatal exposure	299	0.03 [−0.50, 0.57]	0.903	152	0.32 [−0.45, 1.08]	0.416
Postnatal exposure	203	0.01 [−0.70, 0.73]	0.970		0.07 [−0.71, 0.86]	0.855
XeI (hPa s L^−1^)						
Antenatal exposure	299	0.02 [−0.29, 0.32]	0.906	152	0.15 [−0.27, 0.57]	0.472
Postnatal exposure	203	0.37 [−0.03, 0.76]	0.067		0.36 [−0.06, 0.79]	0.096
Rmean (hPa s L^−1^)						
Antenatal exposure	299	0.01 [−0.62, 0.64]	0.979	152	0.38 [−0.52, 1.28]	0.409
Postnatal exposure	203	0.30 [−0.52, 1.13]	0.470		0.60 [−0.33, 1.52]	0.202
*X*mean (hPa s L^−1^)						
Antenatal exposure	299	−0.08 [−0.42, 0.26]	0.633	152	0.02 [−0.49, 0.52]	0.951
Postnatal exposure	203	0.25 [−0.20, 0.70]	0.276		0.05 [−0.46, 0.57]	0.838
ΔR (hPa s L^−1^)						
Antenatal exposure	299	0.19 [−0.20, 0.58]	0.346	152	0.18 [−0.34, 0.70]	0.506
Postnatal exposure	203	0.29 [−0.17, 0.76]	0.217		**0.53 [−0.01, 1.06]**	**0.052**
Δ*X* (hPa s L^−1^)						
Antenatal exposure	299	−0.15 [−0.49, 0.19]	0.390	152	−0.17 [−0.60, 0.25]	0.423
Postnatal exposure	203	−0.16 [−0.55, 0.23]	0.408		−0.36 [−0.80, 0.07]	0.103
FRC (L)						
Antenatal exposure	342	0.01 [−0.01, 0.03]	0.349	191	0.01 [−0.02, 0.03]	0.604
Postnatal exposure	243	−0.02 [−0.04, 0.00]	0.100		−0.02 [−0.04, 0.01]	0.199
LCI (number of turnovers)						
Antenatal exposure	342	**−0.35 [−0.61, −0.09]**	**0.009**	191	**−0.49 [−0.84, −0.14]**	**0.006**
Postnatal exposure	243	0.24 [−0.08, 0.55]	0.138		0.15 [−0.21, 0.50]	0.421
Respiratory rate (min^−1^)						
Antenatal exposure	340	0.07 [−1.10, 1.24]	0.910	184	0.02 [−1.59, 1.62]	0.981
Postnatal exposure	236	1.42 [−0.07, 2.91]	0.062		0.70 [−0.96, 2.35]	0.408
Tidal volume (mL)						
Antenatal exposure	346	−3.76 [−9.77, 2.24]	0.218	187	−7.87 [−16.29, 0.55]	0.067
Postnatal exposure	239	1.62 [−6.51, 9.75]	0.696		1.35 [−7.31, 10.01]	0.759
*t*_E_/*t*_TOT_						
Antenatal exposure	346	−0.27 [−0.99, 0.45]	0.461	187	0.12 [−0.90, 1.15]	0.812
Postnatal exposure	239	0.01 [−0.90, 0.92]	0.980		0.07 [−0.99, 1.13]	0.899
*t*_PTEF_/*t*_E_						
Antenatal exposure	339	−0.50 [−3.12, 2.12]	0.708	182	−0.46 [−4.06, 3.14]	0.802
Postnatal exposure	234	2.19 [−1.18, 5.56]	0.201		2.44 [−1.28, 6.16]	0.197

*Note*: Adjusted model: model of the effect of ante‐ and postnatal exposure to PM10, adjusted for enrolment site, sex, socioeconomic status, BMI *z*‐score, and previous lower respiratory tract illness (LRTI). PM_10_, particulate matter size 10 μg/m^3^; ReE, resistance at end‐expiration; XeE, reactance at end‐expiration; ReI, resistance at end inspiration; XeI, reactance at end inspiration, Rmean, mean resistance; Xmean, mean reactance; ΔR, ReE‐ReI; Δ*X*, XeE‐XeI, FRC, functional residual capacity; LCI, lung clearance index; *t*_*E*_ */t*_*TOT*_, ratio time of expiration to total time; *t*_PTEF_/*t*_E_, ratio time of peak total expiratory flow to time of expiration.

#### VOCs (benzene and/or toluene)

Associations between exposure to VOCs and lung function measures are presented in Table 3. In unadjusted analyses, antenatal exposure to VOCs above ambient standards were associated with increased FRC (*p* = 0.035) and decreased *t*_E_/*t*_TOT_ (*p* = 0.033). Although not statistically significant, antenatal exposure was also associated with lower tidal volume (*p* = 0.063) and higher ReI (*p* = 0.067). In addition, postnatal exposure to VOCs above ambient standards was associated with a decrease in resistance (ReE, ReI and Rmean), as well as an increased reactance (XeI) and RR in unadjusted analyses.

**TABLE 3:** Impact of ante‐ and postnatal exposure to benzene and/or toluene above ambient standards (vs. below ambient standards for both) on lung function at 3 years of age.

	Unadjusted models			Adjusted models		
	*n*	*β* [95% CI]	*p*‐value	*n*	*β* [95% CI]	*p*‐value
ReE (hPa s L^−1^)						
Antenatal exposure	293	0.29 [−0.36, 0.94]	0.378	135	0.35 [−0.63, 1.32]	0.487
Postnatal exposure	181	**−1.12 [−2.01, −0.22]**	**0.015**		**−1.31 [−2.36, −0.26]**	**0.014**
XeE (hPa s L^−1^)						
Antenatal exposure	293	−0.01 [−0.41, 0.40]	0.973	135	0.23 [−0.41, 0.86]	0.482
Postnatal exposure	181	0.45 [−0.11, 1.01]	0.112		0.62 [−0.06, 1.30]	0.073
ReI (hPa s L^−1^)						
Antenatal exposure	293	0.49 [−0.04, 1.02]	0.067	135	0.45 [−0.37, 1.28]	0.276
Postnatal exposure	181	**−0.79 [−1.58, 0.00]**	**0.049**		**−0.88 [−1.75, 0.00]**	**0.050**
XeI (hPa s L^−1^)						
Antenatal exposure	293	0.00 [−0.30, 0.30]	0.991	135	0.28 [−0.16, 0.72]	0.209
Postnatal exposure	181	**0.45 [0.03, 0.88]**	**0.037**		0.31 [−0.16, 0.77]	0.197
Rmean (hPa s L^−1^)						
Antenatal exposure	293	0.47 [−0.16, 1.09]	0.141	135	0.58 [−0.36, 1.53]	0.226
Postnatal exposure	181	**−0.92 [−1.83, −0.02]**	**0.046**		**−1.10 [−2.11, −0.09]**	**0.033**
*X*mean (hPa s L^−1^)						
Antenatal exposure	293	−0.04 [−0.38, 0.30]	0.818	135	0.12 [−0.41, 0.64]	0.658
Postnatal exposure	181	0.41 [−0.08, 0.90]	0.099		0.44 [−0.12, 1.00]	0.120
ΔR (hPa s L^−1^)						
Antenatal exposure	293	−0.20 [−0.59, 0.18]	0.303	135	−0.11 [−0.69, 0.47]	0.711
Postnatal exposure	181	−0.33 [−0.86, 0.21]	0.230		−0.43 [−1.05, 0.19]	0.170
Δ*X* (hPa s L^−1^)						
Antenatal exposure	293	−0.01 [−0.35, 0.33]	0.960	135	−0.05 [−0.53, 0.42]	0.824
Postnatal exposure	181	0.00 [−0.46, 0.46]	0.997		0.31 [−0.19, 0.82]	0.223
FRC (L)						
Antenatal exposure	331	**0.02 [0.00, 0.04]**	**0.035**	173	0.01 [−0.01, 0.04]	0.362
Postnatal exposure	220	0.01 [−0.02, 0.03]	0.503		0.00 [−0.03, 0.02]	0.751
LCI (number of turnovers)						
Antenatal exposure	331	−0.11 [−0.36, 0.15]	0.416	173	0.05 [−0.32, 0.43]	0.834
Postnatal exposure	220	0.04 [−0.31, 0.38]	0.839		0.08 [−0.31, 0.47]	0.697
Respiratory rate (min^−1^)						
Antenatal exposure	328	0.97 [−0.24, 2.17]	0.114	165	1.24 [−0.47, 2.96]	0.103
Postnatal exposure	210	**1.65 [0.02, 3.27]**	**0.047**		**1.85 [0.08, 3.62]**	**0.041**
Tidal volume (mL)						
Antenatal exposure	334	−5.81 [−11.94, 0.32]	0.063	169	−6.34 [−15.39, 2.71]	0.168
Postnatal exposure	214	−0.56 [−9.20, 8.08]	0.898		−1.89 [−11.25, 7.47]	0.690
*t*_E_/*t*_TOT_						
Antenatal exposure	334	**−0.81 [−1.56, −0.06]**	**0.033**	169	−0.58 [−1.64, 0.48]	0.280
Postnatal exposure	214	−0.26 [−1.19, 0.68]	0.591		−0.62 [−1.71, 0.48]	0.267
*t*_PTEF_/*t*_E_						
Antenatal exposure	329	−0.56 [−3.27, 2.15]	0.683	164	−0.91 [−4.69, 2.87]	0.635
Postnatal exposure	208	−1.18 [−4.92, 2.56]	0.535		−1.06 [−4.99, 2.87]	0.596

*Note*: Adjusted model, model of the effect of ante‐ and postnatal exposure to benzene and/or toluene, adjusted for enrolment site, sex, socioeconomic status, BMI *z*‐score, and previous LRTI. ReE, resistance at end‐expiration; XeE, reactance at end‐expiration; ReI, resistance at end inspiration; XeI, reactance at end inspiration, Rmean, mean resistance; *X*mean, mean reactance; ΔR, ReE‐ReI; Δ*X*, XeE‐XeI, FRC, functional residual capacity; LCI, lung clearance index; *t*_*E*_ */t*_*TOT*_, ratio time of expiration to total time; *t*_PTEF_/*t*_E_, ratio time of peak total expiratory flow to time of expiration.

In adjusted analyses, none of the associations between antenatal exposure to VOCs and lung function measures persisted. However, postnatal exposure remained significantly associated with decreased ReE (*p* = 0.014), ReI (*p* = 0.050) and Rmean (*p* = 0.033), and an increased RR (*p* = 0.041) after adjustment for antenatal exposure and confounding.

When adjusting for 6‐week lung function, however, no significant associations were observed between postnatal exposure to VOCs and lung function at 3 years of age (Supporting Information Table S6).

#### ETS

Table 4 presents the impact of ETS exposure on lung function. In unadjusted analyses, antenatal exposure to high levels of ETS was associated with decreased tidal volume (*p* = 0.074), and postnatal exposure with higher ReE (*p* = 0.026) and Rmean (*p* = 0.087). In addition, both moderate and high exposure to postnatal ETS were associated with decreased *t*_PTEF_/*t*_E_ in unadjusted analyses (*p* = 0.066 and *p* = 0.067, respectively). None of these associations persisted in adjusted analyses, or after adjustment for 6‐week lung function (Supporting Information Table S7).

**TABLE 4:** Impact of ante‐ and postnatal exposure to moderate and high levels of tobacco smoke, versus low levels, on lung function at 3 years of age.

	Unadjusted models			Adjusted models		
	*n*	*β* [95% CI]	*p*‐value	*n*	*β* [95% CI]	*p*‐value
ReE (hPa s L^−1^)						
Antenatal exposure				370		
Moderate	429	−0.11 [−0.87, 0.65]	0.775		−0.37 [−1.20, 0.47]	0.387
High		0.34 [−0.44, 1.13]	0.389		−0.37 [−1.35, 0.60]	0.453
Postnatal exposure						
Moderate	379	0.17 [−0.50, 0.84]	0.628		0.02 [−0.75, 0.78]	0.964
High		**1.27 [0.15, 2.39]**	**0.026**		0.70 [−0.62, 2.03]	0.298
XeE (hPa s L^−1^)						
Antenatal exposure				370	−0.16 [−0.67, 0.34]	0.529
Moderate	429	−0.16 [−0.62, 0.30]	0.493			
High		−0.09 [−0.56, 0.39]	0.724		−0.03 [−0.63, 0.56]	0.918
Postnatal exposure					0.16 [−0.30, 0.63]	0.494
Moderate	379	0.12 [−0.28, 0.53]	0.555			
High		0.01 [−0.67, 0.68]	0.988		0.01 [−0.80, 0.82]	0.981
ReI (hPa s L^−1^)						
Antenatal exposure				370		
Moderate	429	0.17 [−0.48, 0.81]	0.614		−0.03 [−0.74, 0.67]	0.925
High		0.36 [−0.31, 1.03]	0.293		−0.14 [−0.97, 0.69]	0.739
Postnatal exposure						
Moderate	379	0.46 [−0.11, 1.02]	0.115		0.34 [−0.31, 0.99]	0.299
High		0.73 [−0.22, 1.68]	0.130			
					0.28 [−0.85, 1.40]	0.631
XeI (hPa s L^−1^)						
Antenatal exposure				370		
Moderate	429	−0.12 [−0.49, 0.25]	0.527			
					−0.04 [−0.45, 0.37]	0.855
High		−0.03 [−0.41, 0.36]	0.894			
					0.03 [−0.45, 0.51]	0.909
Postnatal exposure					−0.09 [−0.46, 0.29]	0.647
Moderate	379	−0.08 [−0.41, 0.25]	0.628			
High		−0.04 [−0.59, 0.50]	0.876		0.02 [−0.64, 0.67]	0.963
Rmean (hPa s L^−1^)						
Antenatal exposure				370		
Moderate	429	−0.13 [−0.87, 0.61]	0.728		−0.34 [−1.15, 0.47]	0.409
					−0.45 [−1.40, 0.50]	0.355
High		0.27 [−0.50, 1.04]	0.486			
Postnatal exposure					0.01 [−0.74, 0.75]	0.986
Moderate	379	0.16 [−0.49, 0.82]	0.626			
High		0.95 [−0.14, 2.04]	0.087		0.38 [−0.91, 1.67]	0.561
Xmean (hPa s L^−1^)						
Antenatal exposure				370	−0.10 [−0.55, 0.35	0.671
Moderate	429	−0.12 [−0.52, 0.29]	0.572			
High		−0.03 [−0.46, 0.39]	0.872		0.08 [−0.45, 0.61]	0.765
Postnatal exposure					0.09 [−0.32, 0.51]	0.657
Moderate	379	0.09 [−0.27, 0.45]	0.617			
High		−0.13 [−0.73, 0.48]	0.682		−0.14 [−0.86, 0.58]	0.701
ΔR (hPa s L^−1^)						
Antenatal exposure				370		
Moderate	429	−0.28 [−0.72, 0.17]	0.226		−0.33 [−0.83, 0.17]	0.192
High		−0.02 [−0.48, 0.45]	0.946		−0.23 [−0.82, 0.35]	0.437
Postnatal exposure						
Moderate	379	−0.29 [−0.69, 0.11]	0.154		‐0.33 [‐0.79, 0.13]	0.164
High		0.54 [−0.13, 1.21]	0.113		0.43 [‐0.37, 1.22]	0.292
ΔX (hPa s L^−1^)						
Antenatal exposure				370		
Moderate	429	−0.04 [−0.43, 0.34]	0.833		−0.12 [−0.54, 0.29]	0.552
High		−0.06 [−0.46, 0.34]	0.768		−0.06 [−0.54, 0.42]	0.810
Postnatal exposure						
Moderate	379	0.20 [−0.13, 0.53]	0.226		0.25 [−0.13, 0.63]	0.193
High		0.05 [−0.50, 0.60]	0.861		−0.01 [−0.66, 0.65]	0.986
FRC (L)						
Antenatal exposure				418		
Moderate	481	0.00 [−0.03, 0.02]	0.700		0.00 [−0.03, 0.02]	0.841
High		−0.02 [−0.04, 0.01]	0.183		−0.01 [−0.04, 0.02]	0.399
Postnatal exposure						
Moderate	428	−0.01 [−0.03, 0.02]	0.586		−0.01 [−0.04, 0.01]	0.368
High		−0.02 [−0.06, 0.02]	0.258			
					−0.03 [−0.08, 0.01]	0.110
LCI (number of turnovers)						
Antenatal exposure				418	0.05 [−0.31, 0.40]	0.796
Moderate	481	−0.03 [−0.34, 0.28]	0.846			
High		−0.01 [−0.33, 0.31]	0.950		0.08 [−0.34, 0.50]	0.711
Postnatal exposure					0.11 [−0.22, 0.43]	0.527
Moderate	428	0.01 [−0.27, 0.29]	0.948			
High		−0.20 [−0.68, 0.27]	0.401		0.03 [−0.54, 0.59]	0.927
Respiratory rate (min^−1^)						
Antenatal exposure				416	1.25 [−0.29, 2.78]	0.111
Moderate	475	0.59 [−0.81, 1.99]	0.407			
High		0.21 [−1.24, 1.65]	0.777		1.60 [−0.22, 3.42]	0.085
Postnatal exposure					0.89 [−0.56, 2.33]	0.231
Moderate	427	0.69 [−0.61, 1.99]	0.297			
High		−1.10 [−3.25, 1.06]	0.317		−0.61 [−3.13, 1.91]	0.637
Tidal volume (mL)						
Antenatal exposure				423		
Moderate	482	−1.63 [−8.65, 5.38]	0.648		−0.33 [−8.02, 7.37]	0.933
High		−6.61 [−13.86, 0.64]	0.074		−1.81 [−10.95, 7.34]	0.698
Postnatal exposure						
Moderate	434	−4.60 [−11.08, 1.88]	0.163		−2.41 [−9.69, 4.87]	0.516
High		−2.74 [−13.50, 8.01]	0.616		5.61 [−7.00, 18.22]	0.382
*t*_E_/*t*_TOT_						
Antenatal exposure				423		
Moderate	482	−0.17 [−0.98, 0.64]	0.686		−0.59 [−1.48, 0.29]	0.188
High		0.03 [−0.80, 0.87]	0.935		−0.43 [−1.49, 0.62]	0.418
Postnatal exposure						
Moderate	434	−0.46 [−1.19, 0.27]	0.213		−0.57 [−1.41, 0.27]	0.180
High		0.36 [−0.85, 1.57]	0.559		−0.09 [−1.54, 1.36]	0.903
*t*_PTEF_/*t*_E_						
Antenatal exposure				417		
Moderate	475	0.00 [−3.05, 3.05]	1.000		1.34 [−1.93, 4.62]	0.421
High		−1.09 [−4.23, 2.05]	0.496		0.33 [−3.55, 4.22]	0.866
Postnatal exposure						
Moderate	428	−2.52 [−5.20, 0.16]	0.066		−1.89 [−4.99, 1.21]	0.231
High		−4.14 [−8.57, 0.28]	0.067		−3.10 [−8.42, 2.22]	0.253

*Note*: Adjusted model: model of the effect of ante‐ and postnatal exposure to tobacco smoke, adjusted for enrolment site, sex, socioeconomic status, BMI *z*‐score, and previous LRTI. ReE: resistance at end‐expiration; XeE: reactance at end‐expiration; ReI: resistance at end inspiration; XeI: reactance at end inspiration, Rmean: mean resistance; Xmean: mean reactance; ΔR: ReE‐ReI; ΔX: XeE‐XeI, FRC: functional residual capacity; LCI: lung clearance index; *t*_E_/*t*_TOT_: ratio time of expiration to total time; *t*_PTEF_/*t*_E_: ratio time of peak total expiratory flow to time of expiration.

## DISCUSSION

In this study, we used comprehensive longitudinal lung function measurements including novel intra‐breath oscillometry, TBFVL and MBW to assess the impact of antenatal and postnatal IAP and ETS exposure on lung function at 3 years of age in an African birth cohort. Adjusting for 6 week lung function allowed us to determine the independent effect of these exposures on postnatal lung function. Both antenatal and postnatal IAP and ETS were associated with decreased lung function at 3 years. Antenatal and postnatal PM_10_ exposure were associated with increased LCI and ΔR, respectively. Antenatal VOC exposure was associated with increased FRC, and both antenatal and postnatal exposure with increased RR. Postnatal ETS exposure was associated with increased ReE.

Antenatal PM_10_ was associated with a decreased LCI. No previous comparative studies have assessed the impact of antenatal PM_10_ exposure using MBW. An Australian study assessed the effect of ambient ultrafine particles (UFP) in 8–11 year old children, and showed no changes in MBW measurements.^26^ This suggests that MBW may not be a sensitive method to assess the effect of PM_10_ on lung function or that PM_10_ exposure antenatally is not associated with long‐term impairment measured by MBW and oscillometry. Also of note, PM_10_ and PM_2.5_ deposit variably within in the airways; PM_10_ in the larger and PM_2.5_ in the smaller airways. Particulate size may hence have different effects on lung growth and function. However, the observed association between postnatal PM_10_ exposure and increased resistance, measured using intra‐breath oscillometry, suggests increased airway obstruction. Similar to our findings, PM_2.5_ exposure was not associated with respiratory impedance measured using oscillometry in 2 year‐old Nigerian children, however, postnatal exposure was associated with lower reactance, suggesting stiffer lungs.^27^ Oscillometry measurements in 8–11 year‐old children similarly showed an association between exposure to UFPs and lower reactance.^26^ Taken together, the results of these studies suggest that postnatal PM exposure impacts negatively on child lung development.^26^, ^27^

The deleterious effects of antenatal ETS exposure on infant lung function and growth are well described.^5^ Here, postnatal exposure to ETS was associated with a decrease in *t*_PTEF_/*t*_E_ and an increase in resistance at 3 years after adjusting for antenatal exposure, which may reflect narrowed airways and predispose a child to recurrent LRTI and possibly chronic lung disease.^7^ These findings are consistent with previous studies which found that postnatal ETS exposure increased oscillometry resistance in children aged 3–14 years.^11^, ^12^ It is difficult to separate the impact of antenatal and postnatal ETS exposure as they are strongly correlated. However, by adjusting for 6‐week lung function we were able to show postnatal effects on lung function in children with continued exposure. This may reflect either an additional effect of postnatal ETS exposure or a persistence of antenatal programming that impairs normal lung development in early life, or likely both.

Antenatal VOC exposure was associated with increased FRC and decreased *t*_E_/*t*_TOT_ at 3 years, suggestive of an obstructive pattern. This extends our prior findings of an association between antenatal benzene exposure and lower expiratory flow ratio, *t*_PTEF_/*t*_E_, at 6 weeks.^9^ Our findings are also in keeping with other studies reporting increased respiratory symptoms and reduced lung function associated with VOC exposure.^3^, ^28^, ^29^ Here, postnatal VOC exposure was further associated with increased RR at 3 years. We observed a paradoxical finding of decreased respiratory system impedance, lower resistance (increased airway calibre) and higher reactance (less stiff lungs). A similar pattern of lower resistance was noted in the study by Robinson et al. assessing the effect of UFP exposure on lung function using oscillometry.^26^ Prior studies of associations between VOC and respiratory outcomes ranged from some effect to no effect and, while increased benzene exposure could impair respiratory health, comparisons between studies are difficult in view of different methodologies.^30^, ^31^ Our study supports altered lung function after VOC exposure, highlighting the importance of avoiding high exposure, but further studies are needed to better understand the impact of VOC exposure on lung development.

This large longitudinal cohort included the measurement of antenatal and postnatal exposures, comprehensive lung function from birth up to 3 years and robust risk factor assessments. There are only a few longitudinal studies which assess antenatal and postnatal exposures and many use spirometry, thus limited to older children. In addition, this is one of the first studies to assess these impacts in a LMIC setting with high prevalence of risk factors for respiratory disease. However, our findings must be considered in light of several limitations. Separating antenatal and postnatal exposures is challenging, but adjusting for 6‐week lung function allowed for the assessment of the independent effects of antenatal and postnatal exposures. However, the sample size precluded a meaningful exploration of potential interactions between antenatal and postnatal exposures. In addition, the small number of children in some of the exposure groups, especially postnatally, limited our ability to detect significant associations, and dichotomising exposure variables reduced statistical power. We measured exposures at two time points, which may underestimate ongoing exposure to IAP and seasonal variations. We conducted multiple comparisons and cannot rule out the possibility of Type 1 errors, however, our findings are in keeping with the existing literature. We included just over half the cohort in analysis and, although the subsample is mostly representative of the entire cohort, differences in HIV exposure, ETS exposure and housing type were observed. In addition, a complete case analysis was used and that this could lead to bias in the results. Further studies and more data are needed to confirm our findings.

Despite some limitations, this study provides novel data suggesting that antenatal IAP and ETS exposures were associated with decreased lung function at 3 years, with postnatal exposures having deleterious effects independent of antenatal exposures. This highlights the need for public health interventions including educational initiatives, especially for women of child‐bearing age to prevent exposure, as well as measures to provide safer, less polluting alternative fuel sources. Our study highlights the vulnerability of children to both antenatal and postnatal IAP and ETS exposure, but further studies are warranted to explore the longer‐term effects of these exposures on lung function and to assess the long‐term clinical implications of these findings.

## AUTHOR CONTRIBUTIONS

**Shaakira Chaya:** Conceptualization (equal); data curation (equal); investigation (equal); writing – original draft (lead); writing – review and editing (equal). **Aneesa Vanker:** Conceptualization (equal); investigation (equal); methodology (equal); writing – review and editing (equal). **Kirsty Brittain:** Formal analysis (lead); writing – review and editing (equal). **Rae Macginty:** Data curation (equal); writing – review and editing (equal). **Carvern Jacobs:** Data curation (equal); investigation (equal); project administration (equal); writing – review and editing (supporting). **Zoltan Hantos:** Data curation (equal); methodology (equal); resources (equal); writing – review and editing (equal). **Heather Zar:** Conceptualization (equal); funding acquisition (equal); supervision (equal); writing – review and editing (equal). **Diane Gray:** Conceptualization (equal); data curation (equal); funding acquisition (equal); investigation (equal); supervision (equal); writing – original draft (equal); writing – review and editing (equal).

## CONFLICT OF INTEREST STATEMENT

D. M. Grey is an Editorial Board member of Respirology and a co‐author of this article. She was excluded from all editorial decision‐making related to the acceptance of this article for publication. No further disclosures were made by the authors.

## HUMAN ETHICS APPROVAL DECLARATION

The study was approved by the University of Cape Town Faculty of Health Sciences human research ethics committee (048/2020; 082/2018; 423/2012). Mothers provided written informed consent in their first language and were reconsented annually.

## Supporting information


**Data S1:** Supporting Information

## Data Availability

The data that support the findings of this study are available from the corresponding author upon reasonable request.
