# Supplementary material for: The impact of antenatal and postnatal indoor air pollution or tobacco smoke exposure on lung function at 3 years in an African birth cohort
Source: Respirology. 2023 Aug 17;28(12):1154–65. doi: 10.1111/resp.14576 (PMC10947154; doi:10.1111/resp.14576)
Supplement: Supplementary file 1 — Data S1: Supporting Information [file RESP-28-1154-s001.docx]

**Supporting Information**

**The impact of indoor air pollution and tobacco smoke exposure on lung function at 3 years of age in an African birth cohort**

Chaya S^1^,Vanker A^1^, MacGinty R^1^, Jacobs C^1^ , Brittain K^1,^ Hantos Z^2^ , Zar HJ^1^ , Gray DM^1^

^1^Department of Paediatrics and Child Health, Red Cross War Memorial Children’s Hospital and SA-MRC Unit on Child and Adolescent Health, University of Cape Town, Cape Town, South Africa; ^2^Department of Anaesthesiology and Intensive Therapy, Semmelweis University, Budapest, Hungary

**Appendix S1- Further Methodological Details**

The Drakenstein Child Health Study is a longitudinal cohort study assessing the impact of risk factors on child health, including environmental, infectious, nutritional, genetic, psychosocial, maternal, and immunological factors. The study area, a peri-urban, low socioeconomic community, is in Paarl, approximately 60 km outside Cape Town, South Africa. Pregnant women were enrolled in their second trimester and followed through childbirth. All births took place at the regional (Paarl) hospital. Birth information was obtained at the time of delivery by study staff members. Visits were synchronised with the national immunisation program, and included visits at 6, 10 and 14 weeks, at 6 and 9 months, and then every 6 months from 12 months onward. Two home visits (one antenatal and one postnatal) were conducted to investigate environmental risk factors, including measurement of indoor air pollution. Anthropometry measurements were recorded at each visit. Robust surveillance for lower respiratory tract infections (LRTI) was undertaken and all events were comprehensively investigated. Study nurses were trained to diagnose LRTI according to the WHO clinical case definitions. Each case of suspected LRTI was examined by study staff and those with confirmed LRTI had a nasopharyngeal swab for respiratory organisms. (1, 2)

**Table S1: Methods and definitions**

VOC: volatile organic compounds, PM_10_ : particulate matter size10 μg/m^3^, LRTI: lower respiratory tract infection

| **Measurement** | **Definition** | **Collected** |
| --- | --- | --- |
| Pollutant measurement equipment |  |  |
| Particulate matter (PM_10_) | personal air sampling pump – SKC Aircheck 52^R^ | -Between 4 and 6 months of life  -All measurements were done in the communal/main living room, away from windows and doors, approximately 1.5 meters from the ground |
| Volatile organic compounds (VOC) benzene and toluene | Markes^R^ thermal desorption tubes using passive diffusion tubes |  |
| National Ambient Air  Quality Standards | Expected exposure for each pollutant based on an averaging period of 1 year for each measure:  -PM_10_: 40 μg/m^3^  -benzene: 5 μg/m^3^  -toluene: 240 μg/m^3^ (3) | -Between 4 to 6 months of life  -VOC: An average concentration based on the 2-week duration in the home was obtained for volatile organic compounds;  -PM_10_: 24-hour averages were obtained |
| Urine cotinine measurement | Urine cotinine tests were performed using the IMMULITER 1000 Nicotine Metabolite Kit (Siemens Medical Solutions Diagnostics^R^, Glyn Rhonwy, United Kingdom).(4) | Antenatal: Maternal urine was collected at the second antenatal study visit and maternal and infant urine at birth. The higher result was used to classify smoking levels.  Postnatal: Infant urine was collected at 6 weeks and yearly |
| Urine cotinine levels | The highest value from two visits:  active smoker (cotinine≥ 500 ng/ml),  passive smoker (10-499 ng/ml ) or  non-smoker (<10 ng/ml).(5) In children these were referred to as high, moderate or no exposure. |  |
| WHO LRTI  case definitions | WHO LRTI case definition: cough or difficulty breathing and age-specific tachypnoea or lower chest wall in-drawing.(6)  WHO severe LRTI case definition: any child under 2 months of age with signs of LRTI or in a child of any age with danger signs (cyanosed, unable to drink, seizures, or decreased level of consciousness).(6, 7) |  |
| Surveillance for LRTI | Active surveillance for LRTI in the cohort was undertaken using community field workers, a short message system (SMS) phone system, ongoing monitoring of cases at health facilities and study staff who could always be contacted.(2) |  |
| Recurrent LRTI | Recurrent LRTI: 2 or more episodes of LRTI in the past 12 months |  |
| Gestational age | Assessed by antenatal ultrasound in the second trimester, if this was unavailable then symphysis-fundal height, recorded  by trained clinical staff at enrolment, or maternal recall of last menstrual period was used (8) | Antenatal 2nd trimester ultrasound/examination |

**Table S2: Comparison of baseline characteristics between children included in and excluded from analysis**

|  | Total cohort | Included in analysis | Not included in analysis ^1^ | P-value |
| --- | --- | --- | --- | --- |
| Number of children | 1,143 | 584 | 559 |  |
| Enrolment site  Mbekweni  TC Newman | 634 (55%)  509 (45%) | 288 (49%)  296 (51%) | 346 (62%)  213 (38%) | <0.001 |
| Mean (SD) maternal age at enrolment | 26.6 (5.7) | 26.8 (5.7) | 26.4 (5.6) | 0.200 |
| Maternal education  Less than secondary  Secondary/any tertiary | 695 (61%)  448 (39%) | 358 (61%)  226 (39%) | 337 (60%)  222 (40%) | 0.725 |
| Housing type: Informal shack | 419 (37%) | 189 (33%) | 230 (41%) | 0.002 |
| Parent employed | 580 (51%) | 300 (51%) | 280 (50%) | 0.665 |
| Household income per month (South African Rand)  <R1000/m  R1000-5000/m  >R5000/m | 386 (34%)  596 (52%)  160 (14%) | 199 (34%)  305 (52%)  80 (14%) | 187 (34%)  291 (52%)  80 (14%) | 0.946 |
| Male sex | 586 (51%) | 289 (49%) | 297 (53%) | 0.218 |
| Delivery mode: Caesarean section | 230 (20%) | 118 (20%) | 112 (20%) | 0.996 |
| Gestation at delivery  <32 weeks  ≥32 and <37 weeks  ≥37 weeks | 33 (3%)  159 (14%)  951 (83%) | 0 (0%)  74 (13%)  510 (87%) | 33 (6%)  85 (15%)  441 (79%) | <0.001 |
| Mean (SD) birth weight z-score | -0.3 (1.1) | -0.3 (1.1) | -0.3 (1.1) | 0.446 |
| Mean (SD) birth length z-score | 0.4 (1.6) | 0.4 (1.6) | 0.4 (1.6) | 0.635 |
| HIV-exposed | 248 (22%) | 110 (19%) | 138 (25%) | 0.016 |
| ***Antenatal exposure to indoor air pollutants above ambient standards*** | | | | |
| PM_10_ (n=767) | 345 (45%) | 179 (44%) | 166 (46%) | 0.599 |
| Benzene (n=738) | 334 (45%) | 170 (43%) | 164 (48%) | 0.244 |
| Toluene (n=738) | 67 (9%) | 33 (8%) | 34 (10%) | 0.429 |
| Benzene and/or Toluene (n=738) | 336 (46%) | 171 (44%) | 165 (48%) | 0.240 |
| ***Exposure to Antenatal tobacco smoke*** | | | | |
| Antenatal exposure (n=1,093):  No exposure  Moderate exposure  High exposure | 249 (23%)  491 (45%)  353 (32%) | 102 (18%)  262 (46%)  202 (36%) | 147 (28%)  229 (43%)  151 (29%) | <0.001 |

^1^ Includes children who did not attend 3-year visit, and children who attended visit but had no successful lung function tests; PM_10_ :particulate matter size10 μg/m^3^

|  | 6 weeks | | 3 years | |
| --- | --- | --- | --- | --- |
|  | n with data | Mean (SD) | n with data | Mean (SD) |
| ReE (hPa.s.L^-1^) | 434 | 45.56 (12.67) | 439 | 12.36 (2.85) |
| XeE (hPa.s.L^-1^) | 434 | -8.23 (8.22) | 439 | -2.63 (1.72) |
| ReI (hPa.s.L^-1^) | 434 | 40.60 (13.67) | 439 | 10.86 (2.43) |
| XeI (hPa.s.L^-1^) | 434 | -5.30 (5.13) | 439 | -3.22 (1.38) |
| Rmean (hPa.s.L^-1^) | 434 | 53.91 (18.50) | 439 | 12.77 (2.79) |
| Xmean (hPa.s.L^-1^) | 434 | -8.96 (7.92) | 439 | -3.45 (1.52) |
| ΔR (hPa.s.L^-1^) | 434 | 4.96 (7.65) | 439 | 1.49 (1.68) |
| ΔX (hPa.s.L^-1^) | 434 | -2.93 (7.37) | 439 | 0.60 (1.43) |
| FRC (L) | 572 | 0.08 (0.02) | 495 | 0.46 (0.09) |
| LCI (number of turnovers) | 572 | 7.15 (0.44) | 495 | 8.23 (1.24) |
| Respiratory rate (min^-1^) | 608 | 48.89 (11.30) | 491 | 27.92 (5.63) |
| Tidal volume (mL) | 608 | 34.75 (5.96) | 498 | 179.23 (28.65) |
| t_E_/t_TOT_ | 608 | 54.98 (4.51) | 498 | 57.78 (3.30) |
| t_PTEF_/t_E_ | 608 | 38.10 (12.22) | 490 | 42.96 (12.20) |

**Table S3: Oscillometry and multiple-breath washout measures at 6 weeks and 3 years of age**

ReE: resistance at end expiration; XeE: reactance at end expiration; ReI: resistance at end inspiration; XeI: reactance at end inspiration, Rmean: mean Resistance; Xmean: mean reactance; ΔR: ReE-ReI; ΔX: XeE-XeI, FRC: functional residual capacity; LCI: Lung clearance index; t_E_/t_TOT_: Ratio time of Expiration to Total time; t_PTEF_/t_E_: Ratio time of Peak Total Expiratory Flow to time of Expiration

|  | ***Antenatal***  ***PM_10_*** | ***Postnatal PM_10_*** | ***Antenatal Benzene*** | ***Postnatal Benzene*** | ***Antenatal Toluene*** | ***Postnatal Toluene*** | ***Antenatal cotinine*** | ***Postnatal cotinine*** |
| --- | --- | --- | --- | --- | --- | --- | --- | --- |
| ***Antenatal PM_10_*** | - |  |  |  |  |  |  |  |
| ***Postnatal PM_10_*** | 0.013 | - |  |  |  |  |  |  |
| ***Antenatal Benzene*** | -0.010 | -0.024 | - |  |  |  |  |  |
| ***Postnatal Benzene*** | 0.008 | 0.117 | -0.020 | - |  |  |  |  |
| ***Antenatal Toluene*** | 0.027 | -0.028 | 0.500 * | -0.068 | - |  |  |  |
| ***Postnatal Toluene*** | -0.012 | 0.218 * | 0.012 | 0.720 * | -0.043 | - |  |  |
| ***Antenatal cotinine*** | -0.084 | 0.090 | -0.005 | -0.017 | -0.011 | 0.060 | - |  |
| ***Postnatal cotinine*** | -0.030 | 0.121 | -0.009 | -0.013 | 0.018 | 0.114 | 0.618 * | - |

**Table S4: Correlation matrix for the different exposures, among children with successful lung function tests**

PM_10_: particulate matter size 10ug/m^3^; * p<0.05

| **Table S5: Impact of postnatal exposure to PM_10_ above ambient standards on lung function at 3 years of age** | | | | | | |
| --- | --- | --- | --- | --- | --- | --- |
|  | Unadjusted models | | | Adjusted model | | |
|  | n | β [95% CI] | P-value | n | β [95% CI] | P-value |
|  | | | | | | |
| ReE (hPa.s.L^-1^) | 203 | 0.31 [-0.51, 1.12] | 0.457 | 119 | 0.64 [-0.35, 1.63] | 0.205 |
| XeE (hPa.s.L^-1^) | 203 | 0.20 [-0.31, 0.71] | 0.435 | 119 | 0.14 [-0.50, 0.77] | 0.672 |
| ReI (hPa.s.L^-1^) | 203 | 0.01 [-0.70, 0.73] | 0.970 | 119 | 0.62 [-0.27, 1.51] | 0.168 |
| XeI (hPa.s.L^-1^) | 203 | 0.37 [-0.03, 0.76] | 0.067 | 119 | 0.00 [-0.52, 0.52] | 0.999 |
| Rmean (hPa.s.L^-1^) | 203 | 0.30 [-0.52, 1.13] | 0.470 | 119 | 0.78 [-0.22, 1.79] | 0.125 |
| Xmean (hPa.s.L^-1^) | 203 | 0.25 [-0.20, 0.70] | 0.276 | 119 | 0.05 [-0.48, 0.58] | 0.854 |
| ΔR (hPa.s.L^-1^) | 203 | 0.29 [-0.17, 0.76] | 0.217 | 119 | -0.03 [-0.61, 0.55] | 0.916 |
| ΔX (hPa.s.L^-1^) | 203 | -0.16 [-0.55, 0.23] | 0.408 | 119 | 0.20 [-0.32, 0.72] | 0.438 |
| FRC (L) | 243 | -0.02 [-0.04, 0.00] | 0.100 | 189 | -0.02 [-0.05, 0.00] | 0.092 |
| LCI (number of turnovers) | 243 | 0.24 [-0.08, 0.55] | 0.138 | 189 | 0.25 [-0.11, 0.62] | 0.170 |
| Respiratory rate (min^-1^) | 236 | 1.42 [-0.07, 2.91] | 0.062 | 199 | 0.77 [-0.70, 2.25] | 0.300 |
| Tidal volume (mL) | 239 | 1.62 [-6.51, 9.75] | 0.696 | 202 | 1.05 [-7.48, 9.59] | 0.808 |
| t_E_/t_TOT_ | 239 | 0.01 [-0.90, 0.92] | 0.980 | 202 | -0.14 [-1.15, 0.88] | 0.792 |
| t_PTEF_/t_E_ | 234 | 2.19 [-1.18, 5.56] | 0.201 | 198 | 1.24 [-2.38, 4.86] | 0.500 |
| Adjusted model: adjusted for enrolment site, sex, socioeconomic status, BMI z-score, previous LRTI, and lung function parameter at 6 weeks of age. | | | | | | |

PM_10_: particulate matter size10ug/m^3^; ReE: resistance at end expiration; XeE: reactance at end expiration; ReI: resistance at end inspiration; XeI: reactance at end inspiration, Rmean: mean Resistance; Xmean: mean reactance; ΔR: ReE-ReI; ΔX: XeE-XeI, FRC: functional residual capacity; LCI: Lung clearance index; t_E_/t_TOT_: Ratio time of Expiration to Total time; t_PTEF_/t_E_: Ratio time of Peak Total Expiratory Flow to time of Expiration

| **Table S6: Impact of postnatal exposure to Benzene and/or Toluene above ambient standards (versus below ambient standards for both) on lung function at 3 years of age** | | | | | | |  |
| --- | --- | --- | --- | --- | --- | --- | --- |
|  | Unadjusted models | | | Adjusted model 2 | | | |
|  | n | β [95% CI] | P-value | n | β [95% CI] | P-value | |
|  | | | | | | |  |
| ReE (hPa.s.L^-1^) | 181 | **-1.12 [-2.01, -0.22]** | **0.015** | 107 | -1.01 [-2.16, 0.14] | 0.085 | |
| XeE (hPa.s.L^-1^) | 181 | 0.45 [-0.11, 1.01] | 0.112 | 107 | 0.48 [-0.25, 1.22] | 0.196 | |
| ReI (hPa.s.L^-1^) | 181 | **-0.79 [-1.58, 0.00]** | **0.049** | 107 | -0.58 [-1.62, 0.47] | 0.279 | |
| XeI (hPa.s.L^-1^) | 181 | **0.45 [0.03, 0.88]** | **0.037** | 107 | 0.54 [-0.03, 1.10] | 0.062 | |
| Rmean (hPa.s.L^-1^) | 181 | **-0.92 [-1.83, -0.02]** | **0.046** | 107 | -0.75 [-1.91, 0.42] | 0.205 | |
| Xmean (hPa.s.L^-1^) | 181 | 0.41 [-0.08, 0.90] | 0.099 | 107 | 0.46 [-0.12, 1.03] | 0.117 | |
| Δ R (hPa.s.L^-1^) | 181 | -0.33 [-0.86, 0.21] | 0.230 | 107 | -0.44 [-1.15, 0.28] | 0.228 | |
| Δ X (hPa.s.L^-1^) | 181 | 0.00 [-0.46, 0.46] | 0.997 | 107 | -0.04 [-0.68, 0.60] | 0.908 | |
| FRC (L) | 220 | 0.01 [-0.02, 0.03] | 0.503 | 175 | 0.00 [-0.03, 0.03] | 0.994 | |
| LCI (number of turnovers) | 220 | 0.04 [-0.31, 0.38] | 0.839 | 175 | 0.13 [-0.27, 0.53] | 0.527 | |
| Respiratory rate (min^-1^) | 210 | **1.65 [0.02, 3.27]** | **0.047** | 182 | 1.39 [-0.17, 2.96] | 0.081 | |
| Tidal volume (mL) | 214 | -0.56 [-9.20, 8.08] | 0.898 | 186 | 0.87 [-8.07, 9.81] | 0.848 | |
| t_E_/t_TOT_ | 214 | -0.26 [-1.19, 0.68] | 0.591 | 186 | -0.29 [-1.30, 0.73] | 0.578 | |
| t_PTEF_/t_E_ | 208 | -1.18 [-4.92, 2.56] | 0.535 | 181 | -0.86 [-4.73, 3.01] | 0.662 | |
| Adjusted model: adjusted for enrolment site, sex, socioeconomic status, BMI z-score, previous LRTI, and lung function parameter at 6 weeks of age. | | | | | | |  |

ReE: resistance at end expiration; XeE: reactance at end expiration; ReI: resistance at end inspiration; XeI: reactance at end inspiration, Rmean: mean Resistance; Xmean: mean reactance; ΔR: ReE-ReI; ΔX: XeE-XeI, FRC: functional residual capacity; LCI: Lung clearance index; t_E_/t_TOT_: Ratio time of Expiration to Total time; t_PTEF_/t_E_: Ratio time of Peak Total Expiratory Flow to time of Expiration

| **Table S7: Impact of postnatal exposure to moderate and high levels of tobacco smoke, versus low levels on lung function at 3 years of age** | | | | | | | |  |
| --- | --- | --- | --- | --- | --- | --- | --- | --- |
|  | | Unadjusted models | | | Adjusted model 2 | | | |
|  |  | n | β [95% CI] | P-value | n | β [95% CI] | P-value | |
|  | | | | | | | |  |
| ReE (hPa.s.L^-1^) | Moderate  High | 379 | 0.17 [-0.50, 0.84]  **1.27 [0.15, 2.39]** | 0.628  **0.026** | 237 | -0.32 [-1.16, 0.52]  -0.30 [-1.88, 1.28] | 0.456  0.707 | |
| XeE (hPa.s.L^-1^) | Moderate  High | 379 | 0.12 [-0.28, 0.53]  0.01 [-0.67, 0.68] | 0.555  0.988 | 237 | 0.15 [-0.34, 0.64]  0.54 [-0.39, 1.47] | 0.552  0.256 | |
| ReI (hPa.s.L^-1^) | Moderate  High | 379 | 0.46 [-0.11, 1.02]  0.73 [-0.22, 1.68] | 0.115  0.130 | 237 | 0.07 [-0.68, 0.82]  -0.14 [-1.55, 1.26] | 0.848  0.840 | |
| XeI (hPa.s.L^-1^) | Moderate  High | 379 | -0.08 [-0.41, 0.25]  -0.04 [-0.59, 0.50] | 0.628  0.876 | 237 | 0.08 [-0.35, 0.51]  0.05 [-0.76, 0.86] | 0.702  0.906 | |
| Rmean (hPa.s.L^-1^) | Moderate  High | 379 | 0.16 [-0.49, 0.82]  0.95 [-0.14, 2.04] | 0.626  0.087 | 237 | -0.37 [-1.21, 0.48]  -0.17 [-1.76, 1.42] | 0.394  0.835 | |
| Xmean (hPa.s.L^-1^) | Moderate  High | 379 | 0.09 [-0.27, 0.45]  -0.13 [-0.73, 0.48] | 0.617  0.682 | 237 | 0.19 [-0.25, 0.62]  0.10 [-0.72, 0.92] | 0.401  0.814 | |
| Δ R (hPa.s.L^-1^) | Moderate  High | 379 | -0.29 [-0.69, 0.11]  0.54 [-0.13, 1.21] | 0.154  0.113 | 237 | -0.36 [-0.86, 0.13]  -0.07 [-1.01, 0.86] | 0.147  0.874 | |
| Δ X (hPa.s.L^-1^) | Moderate  High | 379 | 0.20 [-0.13, 0.53]  0.05 [-0.50, 0.60] | 0.226  0.861 | 237 | 0.05 [-0.36, 0.47]  0.38 [-0.41, 1.17] | 0.798  0.345 | |
| FRC (L) | Moderate  High | 428 | -0.01 [-0.03, 0.02]  -0.02 [-0.06, 0.02] | 0.586  0.258 | 326 | -0.01 [-0.04, 0.02]  -0.03 [-0.08, 0.01] | 0.495  0.163 | |
| LCI (number of turnovers) | Moderate  High | 428 | 0.01 [-0.27, 0.29]  -0.20 [-0.68, 0.27] | 0.948  0.401 | 326 | 0.14 [-0.21, 0.49]  0.12 [-0.47, 0.72] | 0.428  0.690 | |
| Respiratory rate(min^-1^) | Moderate  High | 427 | 0.69 [-0.61, 1.99]  -1.10 [-3.25, 1.06] | 0.297  0.317 | 350 | 0.88 [-0.54, 2.31]  -0.64 [-3.10, 1.82] | 0.224  0.607 | |
| Tidal volume (mL) | Moderate  High | 434 | -4.60 [-11.08, 1.88]  -2.74 [-13.50, 8.01] | 0.163  0.616 | 357 | 0.89 [-6.47, 8.25]  8.10 [-4.57, 20.77] | 0.813  0.209 | |
| t_E_/t_TOT_ | Moderate  High | 434 | -0.46 [-1.19, 0.27]  0.36 [-0.85, 1.57] | 0.213  0.559 | 357 | -0.55 [-1.42, 0.32]  -0.06 [-1.55, 1.44] | 0.217  0.942 | |
| t_PTEF_/t_E_ | Moderate  High | 428 | -2.52 [-5.20, 0.16]  -4.14 [-8.57, 0.28] | 0.066  0.067 | 352 | -1.71 [-4.83, 1.42]  -2.36 [-7.71, 2.99] | 0.283  0.386 | |
| Adjusted model: adjusted for enrolment site, sex, socioeconomic status, BMI z-score, previous LRTI, and lung function parameter at 6 weeks of age. | | | | | | | |  |

ReE: resistance at end expiration; XeE: reactance at end expiration; ReI: resistance at end inspiration; XeI: reactance at end inspiration, Rmean: mean Resistance; Xmean: mean reactance; ΔR: ReE-ReI; ΔX: XeE-XeI, FRC: functional residual capacity; LCI: Lung clearance index; t_E_/t_TOT_: Ratio time of Expiration to Total time; t_PTEF_/t_E_: Ratio time of Peak Total Expiratory Flow to time of Expiration

**Figure S1: Directed acyclic graph constructed to select minimum set of adjustment variables**


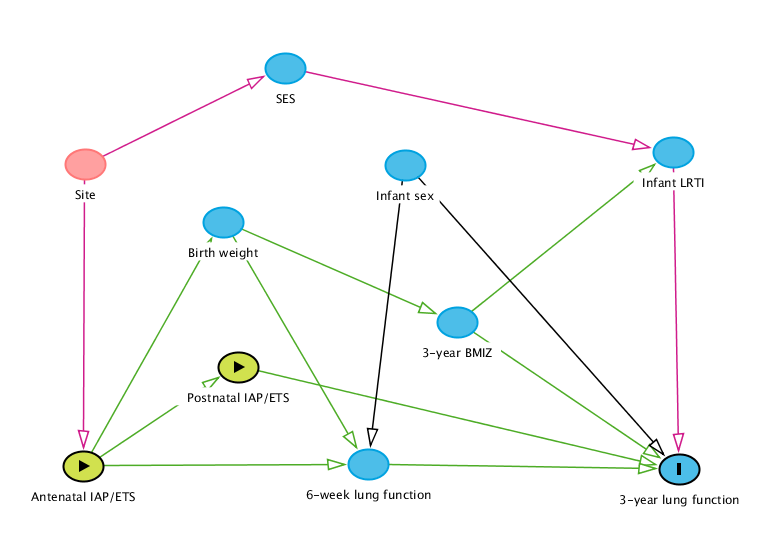


**References**

1. Government Gazette Republic of South Africa. National Ambient Air Quality Standards 2009 [cited 2014 August]. Available from: <https://www.environment.gov.za/sites/default/files/legislations/nemaqa_airquality_g32816gon1210.pdf>.

2. Siemens. Immulite 1000 Nicotine Metabolite. UK, 2009. Available from: https://www.siemens-healthineers.com/en-za/immunoassay/systems/immulite-1000-immunoassay-system.

3. Vanker A, Barnett W, Brittain K, Gie R, Koen N, Myers B, et al. Antenatal and early life tobacco smoke exposure in an African birth cohort study. The International Journal of Tuberculosis and Lung Disease. 2016;20(6):729-37.

4. World Health Organization (WHO). Integrated Management of Childhood Illness: distance learning course. 2014.

5. Zar HJ, Barnett W, Stadler A, Gardner-Lubbe S, Myer L, Nicol MP. Aetiology of childhood pneumonia in a well vaccinated South African birth cohort: a nested case-control study of the Drakenstein Child Health Study. The Lancet Respiratory Medicine. 2016;4(6):463-72.

6. Zar HJ, Pellowski JA, Cohen S, Barnett W, Vanker A, Koen N, et al. Maternal health and birth outcomes in a South African birth cohort study. PloS one. 2019;14(11):e0222399.
